# Pyroptosis related genes signature predicts prognosis and immune infiltration of tumor microenvironment in hepatocellular carcinoma

**DOI:** 10.1186/s12885-022-10097-2

**Published:** 2022-09-20

**Authors:** Guoxu Fang, Qinghua Zhang, Jianhui Fan, Haitao Li, Zongren Ding, Jun Fu, Yijun Wu, Yongyi Zeng, Jingfeng Liu

**Affiliations:** 1 Department of Hepatopancreatobiliary Surgery, Mengchao Hepatobiliary Hospital of Fujian Medical University, Xihong Road 312, Fuzhou, 350025 China; 2 The Big Data Institute of Southeast Hepatobiliary Health Information, Mengchao Hepatobiliary Hospital of Fujian Medical University, Fuzhou, 350025 China; 3 The Graduate School of Fujian Medical University, Fuzhou, 350108 China; 4 Department of Hepatology for Pregnancy, Mengchao Hepatobiliary Hospital of Fujian Medical University, Fuzhou, 350025 China; 5 Department of Hepatopancreatobiliary Surgery, Fujian Medical University Cancer Hospital, Fujian Cancer Hospital, Fuzhou, 350014 China

**Keywords:** HCC, Prognosis, Pyroptosis, Overall survival, Immune infiltration

## Abstract

Little is known on the relationship between the expression of pyroptosis related genes (PRGs) and prognosis of hepatocellular carcinoma (HCC). In this study, a specific PRGs prognostic model was developed with an aim to improve therapeutic efficiency among HCC patients. In total, 42 PRGs that were differentially expressed between HCC tissues and adjacent tissues and we exhibited the mutation frequency, classification, the location of copy number variation (CNV) alteration and the CNV variation frequency of PRGs. Two clusters were distinguished by the consensus clustering analysis based on the 42 differentially expressed genes (DEGs). There were significant differences in clinical features including T stage, grade, gender, and stage among different clusters. Kaplan–Meier curve analysis showed that cluster 1 had a better prognosis than cluster 2. The prognostic value of PRGs for survival was evaluated to construct a multigene signature using The Cancer Genome Atlas (TCGA) cohort. Based on the univariate analysis and multivariate analysis, a 10-gene signature was built and all HCC patients in the TCGA cohort were divided into low-risk group and high-risk group. HCC patients in the high-risk group showed significantly lower survival possibilities than those in the low-risk group (*P* < 0.001). Utilizing the median risk score from the TCGA cohort, HCC patients from International Cancer Genome Consortium (ICGC)-LIRI-JP cohort and Gene Expression Omnibus (GEO) cohort (GSE14520) were divided into two risk subgroups. The result showed that overall survival (OS) time was decreased in the high-risk group. Combined with the clinical characteristics, the risk score was an independent factor for predicting the OS of HCC patients. Then, ROC curve and survival analysis were performed to evaluate the prognostic prediction value of the model. Finally, we constructed a PRGs clinical characteristics nomogram to further predict HCC patient survival probability. There were significant differences in immune cell infiltration, GSEA enrichment pathway, IC50 of chemotherapeutics, PRGs mutation frequency between high-risk group and low-risk group. This work suggests PRGs signature played a crucial role in predicting the prognosis, infiltration of cancer microenvironment, and sensitivity of chemotherapeutic agents.

**Supplementary Information:**

The online version contains supplementary material available at 10.1186/s12885-022-10097-2.

## Introduction

Hepatocellular carcinoma (HCC), one of the most common gastrointestinal cancers, has been considered as a worldwide threat due to a high incidence and poor prognosis. Based on the global cancer statistics in 2018, there were 841,080 new HCC cases and 781,631 deaths [1]. The disease is rapidly progressed, and most patients show a 5-year survival rate of merely 5-14 % [2]. Resistance to apoptotic process has been considered to be closely related to poor prognosis among HCC patients [3]. Therefore, it is necessary to find a new candidate of programmed cell death in order to overcome the drug resistance and develop new models for predicting the overall survival (OS).

Pyroptosis, designated as a novel programmed cell death pathway usually caused by activation of inflammasome and caspase, plays an important role in the progress of HCC [4–8]. Pyroptosis pathways include canonical pyroptotic pathways mediated by caspase-1 dependence and non-canonical pyroptotic pathways mediated by caspase-4, − 5, and − 11. Currently, pyroptosis is reported to involve in the pathogenesis of several cancers through modulating the proliferation, invasion, cell cycle and drug-resistance of cancer cells. In colon cancer cells, the cleavage of GSDME by caspase-3 was crucial for the lobaplatin-induced pyroptosis [9]. In addition, tumor suppressor DRD2 could restrict the restricts NF-kappaB signaling to trigger pyroptosis, which played a pivotal role in the pathogenesis of breast cancer [10]. Moreover, the ROS/NLRP3/caspase-1/GSDMD-mediated pyroptotic pathway was closely related to the inhibition of endometrial cancer growth mediated by hydrogen [11]. All these indicated that pyroptosis may serve as a crucial factor for the pathogenesis of cancer. To date, extensive studies show that pyroptosis is closely linked to the cancer immunity, and may serve as a candidate to improve the prediction efficiency and immune response [12]. Unlike the apoptosis, pyroptosis led to massive release of inflammatory factors, triggering severe immune responses that may involve in the remodeling of the tumor microenvironment [13].

Recently, the potential application of differentially expressed genes have been a new hot in the research of cancer. Therefore, it is beneficial to investigate the potential application of pyroptosis-related genes (PRGs) in the treatment and prognosis of HCC. In this study, we determined the expression of PRGs between the HCC tissues and the adjacent tissues, with an aim to investigate the correlation between pyroptosis and the tumor microenvironment, which could guide the target therapy and immune therapy for HCC patients.

## Materials and methods

### Datasets

The TCGA-liver cancer dataset consisted of the RNA-seq data, somatic mutation data, and copy number variation (CNV) data from 374 liver cancer tissue and 50 adjacent normal samples. Their clinical characteristics were downloaded from the TCGA database. Gene expression profile of GSE14520 was downloaded from the GEO database. The TCGA dataset was enrolled as a training cohort and the GEO dataset was regarded as the external validation cohort. Besides, the ICGC-LIRI-JP cohort was downloaded from the ICGC database also served as external validation cohort. As these data were open-access, therefore, the ethical approval by an ethics committee was not required.

### Identification of differentially expressed pyroptosis related genes (PRGs)

In total, 52 PRGs were extracted from prior publications [14–17], previous pyroptosis-related studies [18–31] and MSigDB database (v7.4), [32], respectively (Supplementary Table S1). The expression data in all the three datasets were normalized to fragment per kilobase million (FPKM) values before comparison. Limma statistical package was used to identify DEGs with a *P* value of less than 0.05. A PPI network for the differentially expressed PRGs was constructed with Search Tool for the Retrieval of Interacting Genes (STRING, version 11.0).

### Unsupervised clustering analysis of PRG

Unsupervised cluster analysis was performed to identify different pyroptosis type using the ConsenSuClusterPlus R package. Patients from TCGA database were divided into different groups for subsequent analysis. A total of 2000 repeats were performed to ensure the stability of the classification. The correlation between different clusters and clinical information was further determined by Chi-square test. The OS of each cluster was performed using the Kaplan–Meier survival curve.

### Development and validation of the PRGs prognostic model

To assess the prognostic value of the PRGs, Cox regression analysis was employed to evaluate the correlations between each gene and survival status in the TCGA cohort. Univariate and multivariate COX analyses were performed on the PRGs. The expression data of the PRGs associated with prognosis in the TGCA dataset were used to establish the risk score model. GSE14520 and ICGC-LIRI-JP were then used to verify the reliability of the model. The risk score was calculated after centralization and standardization of the TCGA expression data, based on the following formula: Risk Score= ${\sum}_i^{10} Xi\times Yi$, with X and Y represented the coefficients and gene expression level, respectively. TCGA HCC patients were divided into low-risk group and high-risk group according to the median risk score. A log-rank test was used to compare the survival difference between the two groups. The OS of each group was analyzed using the Kaplan–Meier survival curve. The 1-year, 3-year and 5-year survival ROC curve was analyzed using the “survival”, “survminer” and “timeROC” R packages.

### Independent prognostic analysis of the risk score

In the prognostic analysis of risk score, we extracted the clinical information from the TCGA cohort, including age, gender, grade, stage, T stage, N stage, and M stage. These variables were analyzed in combination with the risk score in our regression model based on the univariate and multivariable Cox regression models.

### Correlation analysis between immunity and the risk groups

Spearman correlation was used to analyze the correlation between risk score values and tumor-infiltrating immune cells (TIIC) based on XCELL, TIMER, QUANTISEQ, MCPCOUNTER, EPIC, CIBERSORT, and CIBERSORT-ABS algorithms. In addition, the heatmap was utilized to depict the component differences of immunocytes between the high- and low-risk groups.

### Function analysis between high- and low-risk groups

GSEA was used to investigate the biological function of PRGs. To assess the signature in clinical trials for HCC treatment, R ggplot2 and pRRophetic packages were utilized to calculate the lower half inhibitory concentration (IC50) of commonly used chemotherapeutic drugs (e.g. lapatinib) in TCGA-HCC. Moreover, somatic mutations were explored among high- and low-risk groups using maftools, which was an R package for analyzing and visualizing mutation annotation format (MAF) files from large-scale sequencing studies.

### Quantitative reverse transcription PCR (qRT-PCR)

Cell total RNA was extracted using Trizol reagent (Invitrogen, USA) following the manufacturer’s instructions. The quantity and quality of extracted RNA were assessed by the spectrophotometric (Dojindo Laboratories, Kumamoto, Japan) determination of absorbance ratio (A260/A280). Then, the prepared RNA was reversely transcribed into cDNA using reverse transcriptase (Invitrogen, USA) using random primers. One microliter of synthesized cDNA was used in each PCR reaction. The qRT-PCR was conducted using SYBR Green on ABI PRISM 7300HT Sequence Detection System (Applied Biosystems, USA) using specific primers listed in Supplementary Table 2. β-Actin was used as a control for normalization.

### Statistical analysis

Statistical analysis was performed using R software (version 4.0.2). The differences between the groups were compared using the log-rank test. Cox proportional hazard model was used to analyze the significant PRGs affecting OS. *P* < 0.05 was considered to be statistically significant.

## Results

### Differentially expressed PRGs in the TCGA cohort and landscape of genetic and expression variation of PRGs in HCC

**Fig. 1:**
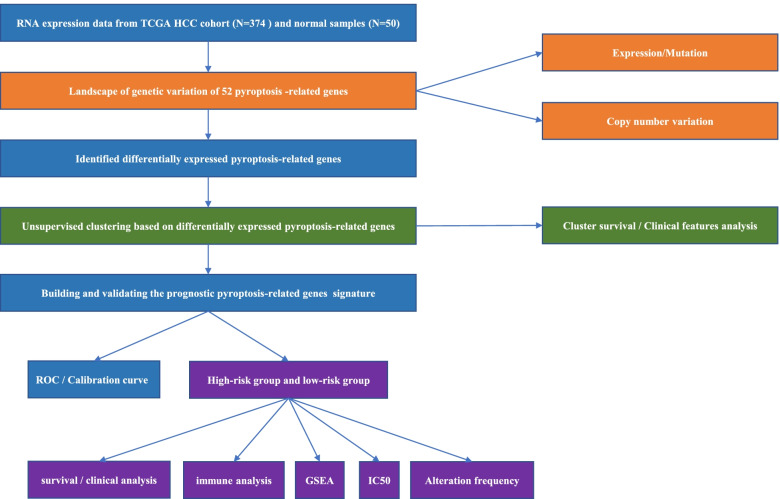
The specific workflow graph for this study

**Fig. 2:**
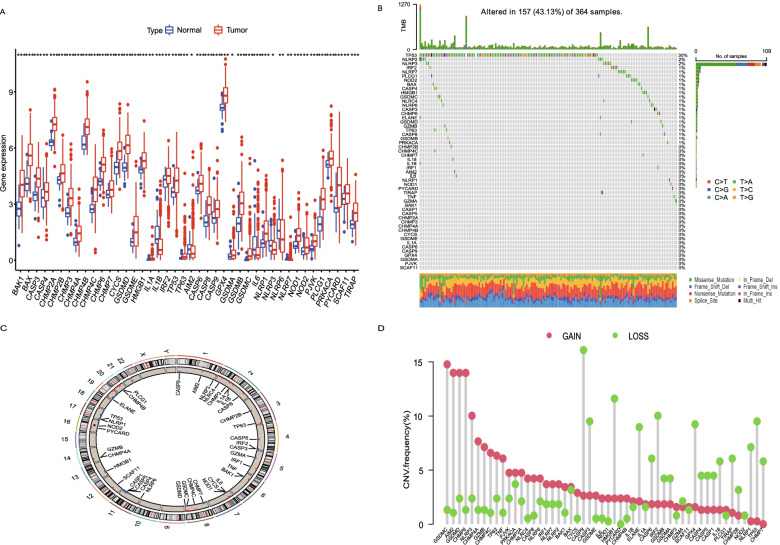
Differentially expressed PRGs in the TCGA cohort and landscape of genetic and expression variation of PRGs in HCC. **A** 42 Differentially expressed PRGs were identified in HCC tissues and adjacent tissues. **B** Mutation frequency and classification of 52 PRGs in HCC. **C** Location of CNV alteration of 52 PRGs on 23 chromosomes in the HCC cohort. **D** CNV variation frequency of 52 PRGs in the HCC cohort. The height of the column represented the alteration frequency. ***P < 0.001, **P < 0.01, *P < 0.05

The flowchart of data analysis was shown in Fig. 1. We compared the expression of 52 PRGs in TCGA data from 50 adjacent tissues and 374 tumor tissues, and finally 42 DEGs were identified. Among these DEGs, 5 genes were down-regulated in tumor group including IL1B, AIM2, IL6, NLRP3, and NLRP6. The other 37 genes were enriched in the tumor group, including BAK1, BAX, CASP3, CASP4, CHMP2A, CHMP2B, CHMP3, CHMP4A, CHMP4B, CHMP4C, CHMP6, CHMP7, CYCS, GSDMD, GSDME, HMGB1, IL1A, IRF2, TP53, TP63, CASP6, CASP8, CASP9, GPX4, GSDMA, GSDMB, GSDMC, NLRP1, NLRP7, NOD1, NOD2, PJVK, PLCG1, PRKACA, PYCARD, SCAF11, and TIRAP (Fig. 2A). We then demonstrated the incidence of CNVs and somatic mutations of 52 PRGs in HCC. As shown in Fig. 2B, genetic mutation was identified in 157 of 364 (43.13%) HCC samples. Missense mutation was the most common variant. In addition, TP53 gene showed the highest mutation frequency, followed by NLRP2 and NLRP3 genes (Fig. 2B). Figure 2C presented the location of CNV alterations of the 52 PRGs on chromosomes. For the CNV alteration frequency, all the 52 PRGs showed prevalent CNV alteration. More than half of the 52 PRGs had copy number amplification, while the CNV deletion frequencies of CASP9, CASP3, HMGB1, ELANE, CASP6, IRF2, GSDMB, GSDMA, GPX4, CASP4, CASP5, CASP1, IL18, TIRAP, CHMP2B, NLRP1, TP53 and CHMP7 were widespread (Fig. 2D).

The PPI network analysis was given to further explore the interactions of these PRGs. As shown in Supplementary Fig. S1A, the minimum interaction score was set at 0.9 with the highest confidence. Seven hub genes were screened including IL1B, NLRP3, PYCARD, CASP8, CASP3, TP53, as well as CHMP2A. The correlation network containing all PRGs was presented in Supplementary Fig. S1B.

### Differentially expressed PRGs between HCC tissues and adjacent Normal tissues

**Fig. 3:**
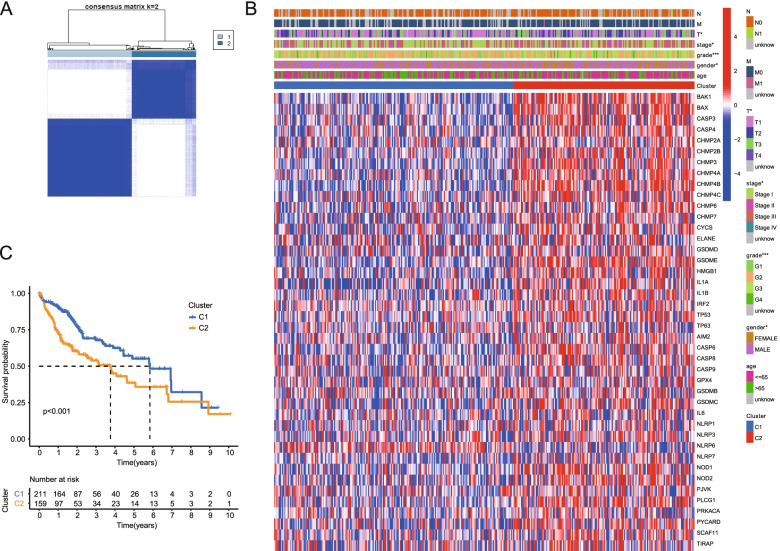
Tumor classification based on the pyroptosis-related DEGs. **A** HCC patients (*n* = 370) were divided into two clusters according to the consensus clustering matrix (k = 2). **B** Heatmap and the clinicopathologic characters of the two clusters classified by these DEGs. **C** Kaplan–Meier OS curves for the two clusters

We found 42 differential PRGs in normal paracancer samples and tumor samples of HCC by analyzing TCGA database. The Volcano plots of DEGs is shown in Supplementary Fig. S2. To explore the correlation between the expression of the 42 DEGs and HCC clusters, we performed a consensus clustering analysis involving 374 HCC patients in the TCGA cohort. When the clustering variable (k) was set at 2, the intragroup correlations were the highest and the intergroup correlations were the lowest, indicating that the 374 HCC patients could be well divided into two clusters based on the 42 DEGs (Fig. 3A). The gene expression profile and the clinical features were presented in a heatmap, which showed significant differences in the distribution of T stage, stage, grade and gender between the two cluster groups (*P* < 0.05). In contrast, there was no statistical difference in the distribution of N stage, M stage and age between the two groups (*P* > 0.05, Fig. 3B). There is a significant difference in the OS time of two clusters (*P* < 0.001, Fig. 3C).

### Development of a prognostic gene model in the TCGA cohort

**Fig. 4:**
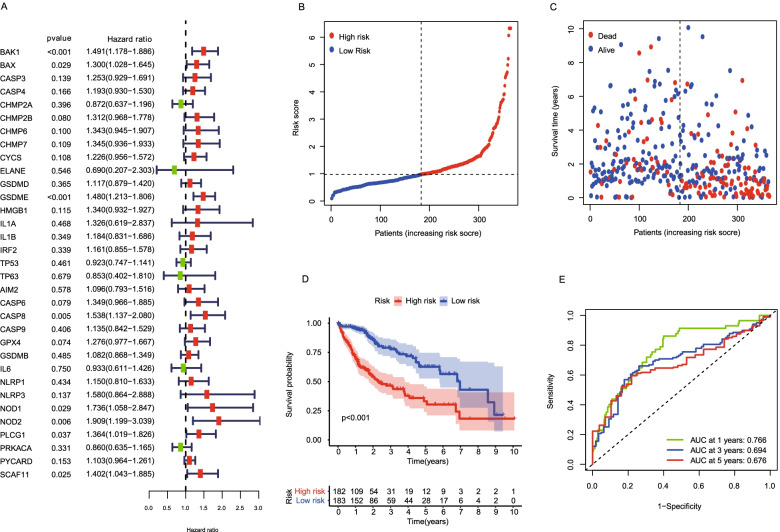
Construction of risk signature in the TCGA cohort. **A** Univariate Cox regression analysis for screening the survival-related pyroptosis genes. **B** Distribution of patients based on the risk score in the TCGA cohort. **C** The survival status for each patient in the TCGA cohort (low-risk population: on the left side of the dotted line; high-risk population: on the right side of the dotted line). **D** Kaplan–Meier curves for the OS of patients in the low-risk group and high-risk group in the TCGA cohort. **E** ROC curves demonstrated the predictive efficiency of the risk score in the TCGA cohort

A total of 374 HCC samples were matched with the corresponding patients with complete survival information. Univariate Cox regression analysis was used to screen the survival-related pyroptosis genes (Fig. 4A). Then, these genes were performed by multivariate Cox regression analysis. In total, 10 genes were identified and used for the subsequent modeling, including BAK1, BAX, CHMP2A, GSDME, IL1A, TP53, TP63, GPX4, PRKACA and SCAF11. The risk score was calculated as follows: risk score = (0.368611051021708 * expression of BAK1) + (0.308688517099686 * expression of BAX) + (− 0.52007432297355 *expression of CHMP2A) + (0.330587747807719 * expression of GSDME) + (− 0.807361948750797 * expression of IL1A) + (− 0.323671479794998 * expression of TP53) + (− 0.604855494168515* expression of TP63) + (0.512445054990862 * expression of GPX4) + (− 0.283264209118667 * expression of PRKACA) + (0.432681763927682 * expression of SCAF11). Based on the median score calculated by the risk score formula, 374 patients were equally divided into low-risk group and high-risk group (Fig. 4B). Patients in the high-risk group showed a higher death rate and a shorter survival time than those in the low-risk group (Fig. 4C). A notable difference in OS time was detected between the low-risk group and high-risk group (*P* < 0.001, Fig. 4D). Time dependent ROC analysis was applied to evaluate the sensitivity and specificity of the prognostic model, yielding AUC of 0.766 for 1-year, 0.694 for 3-year, and 0.676 for 5-year survival, respectively (Fig. 4E).

### External validation of the risk signature

**Fig. 5:**
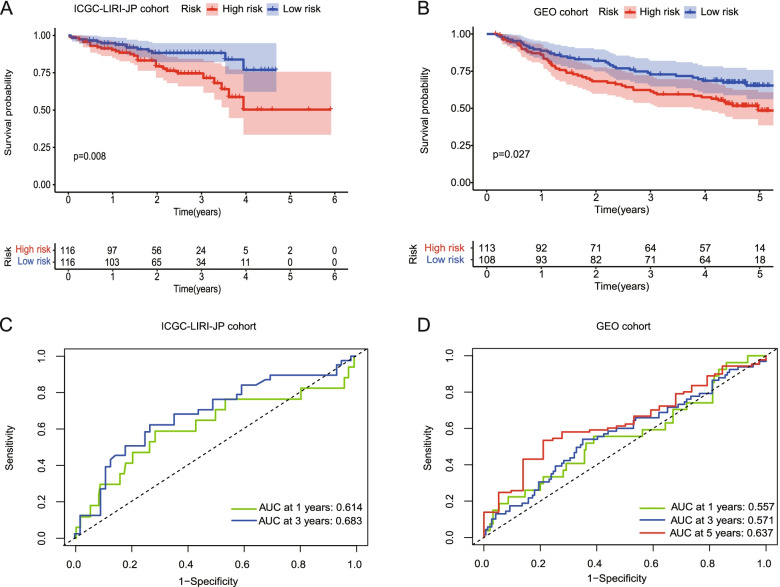
Validation of the risk model in the ICGC cohort and GEO cohort. **A** Kaplan–Meier curves for the OS of patients in the low-risk group and high-risk group in the ICGC cohort. **B** Kaplan–Meier curves for the OS of patients in the low-risk group and high-risk group in the GEO cohort. **C** ROC curves demonstrated the predictive efficiency of the risk score in the ICGC cohort. **D** ROC curves demonstrated the predictive efficiency of the risk score in the GEO cohort

Patients from GSE14520 and ICGC-LIRI-JP cohorts were utilized as the validation set. Kaplan–Meier analysis indicated a significant difference in the survival rate between the low-risk group and high-risk group in the ICGC-LIRI-JP cohort (*P* = 0.008, Fig. 5A), as well as the GSE14520 cohort (*P* = 0.027, Fig. 5B). ROC curve analysis of the ICGC cohort showed that our model had good predictive efficacy for 1-year (AUC = 0.614) and 3-year surivival (AUC = 0.683) (Fig. 5C), respectively. The GSE14520 cohort showed that the model had good predictive efficacy for 3-year survival (AUC = 0.571) and 5-year survival (AUC = 0.637) (Fig. 5D).

### Independent prognostic value of the risk model

**Fig. 6:**
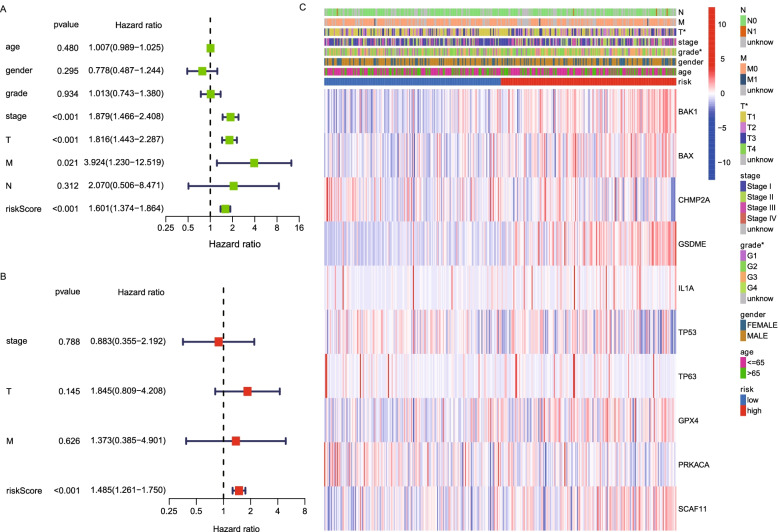
Univariate and multivariate Cox regression analyses for the risk score. **A** Univariate analysis of risk score and clinicopathological characteristics. **B** Multivariate analysis of risk score and clinicopathological characteristics. **C** Heatmap for the connections between clinicopathologic features and the risk groups (*P < 0.05)

Univariate and multivariable Cox regression analyses were performed to evaluate whether the risk score derived from the gene signature model could serve as an independent prognostic factor. The univariate Cox regression analysis indicated that the risk score was an independent factor for poor survival in the TCGA cohort (HR = 1.601, 95% CI: 1.374–1.864, Fig. 6A). The multivariate analysis also implied that, after adjusting for other confounding factors, the risk score was an independent prognostic factor for patients with HCC in the TCGA cohort (HR = 1.485, 95% CI: 1.261–1.750, Fig. 6B). In addition, a heatmap of clinical features for the TCGA cohort indicated that the T stage and grade were differently distributed between the low-risk group and high-risk group (Fig. 6C).

### Establishment and evaluation of a nomogram for predicting patient 1-year, 3-year and 5-year OS

**Fig. 7:**
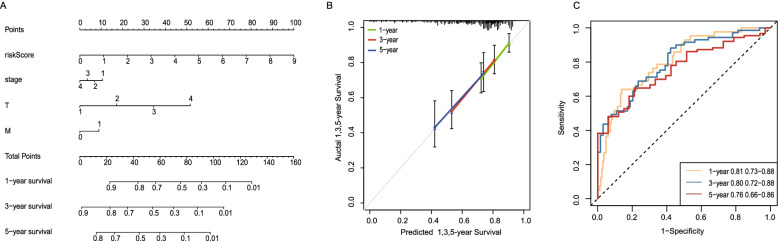
Establishment and evaluation of a nomogram based on the TCGA dataset. **A** A nomogram for predicting 1-year, 3-year and 5-year OS. **B** The calibration curves for predicting 1-year, 3-year and 5-year OS. **C** The areas under the ROC curves for predicting 1-year, 3-year and 5-year OS

Four prognostic factors were combined to establish a nomogram for predicting 1-year, 3-year and 5-year OS based on the TCGA dataset (Fig. 7A). The calibration curves for predicting 1-year, 3-year and 5-year OS were in good agreement with the observed values (Fig. 7B). The AUC for predicting 1-year, 3-year and 5-year OS was 0.81, 0.80 and 0.76, respectively (Fig. 7C).

### Relationship between prognostic signature and immune infiltration

**Fig. 8:**
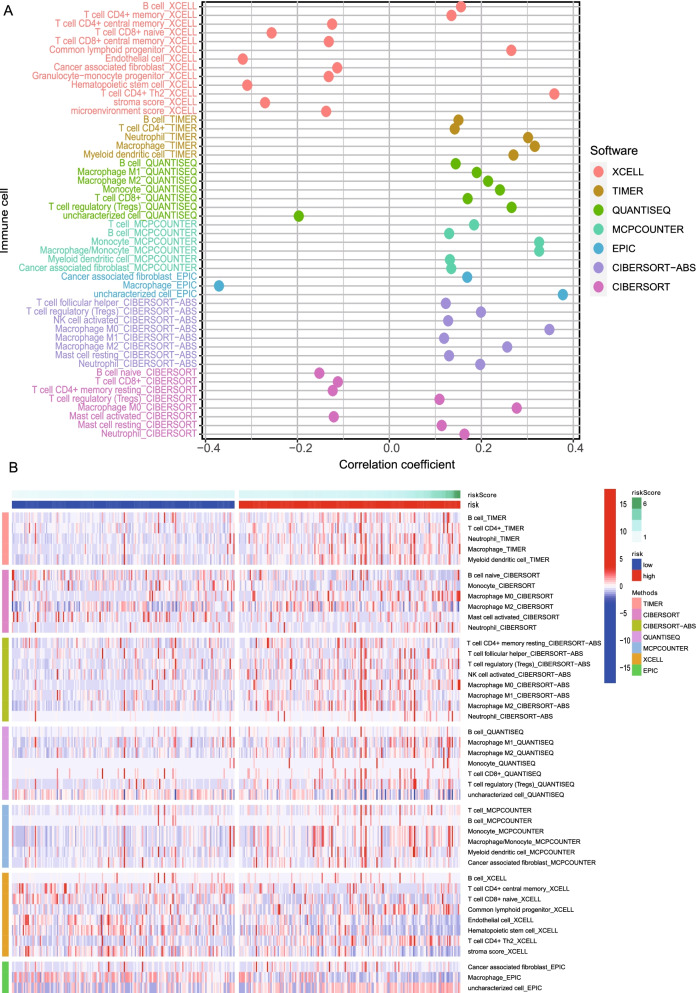
Relationship between prognostic signature and immune infiltration. **A** The correlation between risk score and immune cell infiltration was analyzed by Spearman correlation analysis using different algorithms. **B** The heatmap of immune infiltration based on different algorithms among the high- and low-risk group

Firstly, we examined component differences of immune cells between high- and low-risk groups, as well as risk score values. Spearman correlation analysis was performed using different algorithms, with a resulting lollipop shape, as displayed in Fig. 8A. The results indicated that most immune cells were positively correlated with the risk score, which was consistent with our GSEA finding that the high-risk group was predominantly enriched in immune-related pathways. The heatmap demonstrated that the infiltration of most immune cells was higher in the high-risk group than in the low-risk group (Fig. 8B). We further elucidated the correlation of PRGs expression with each type of immune cell infiltration. The infiltration of NK cells was positively correlated with the expression of CHMP2A, while the infiltration of macrophages M1 was negatively correlated with the GSDME (Supplementary Fig. S3).

### Gene set enrichment analysis (GSEA) and mutation data analysis of PRGs between the high- and low-risk group

**Fig. 9:**
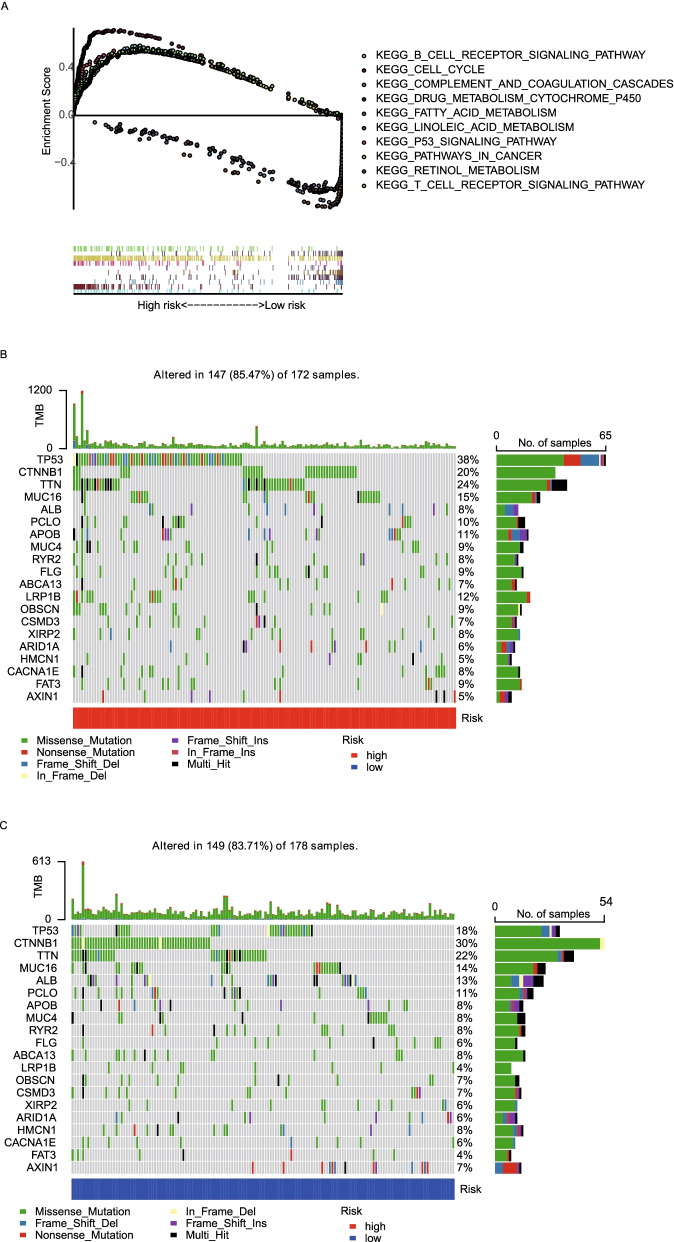
Gene set enrichment analysis (GSEA) and mutation data analysis of PRGs between the high- and low-risk group. **A** GSEA results suggested that the two risk groups were mainly enriched in tumor immunity and metabolism. **B** The top 20 driver genes with the highest alteration in the high-risk group. **C** The top 20 driver genes with the highest alteration in the low-risk group

GSEA had an advantage in exploring the involved signaling pathways, which revealed that the genes in the high-risk group of TCGA cohorts were significantly enriched in tumor and immune-related pathways such as B cell receptor signaling pathway, T cell receptor signaling pathway, P53 signaling pathway, pathways involved in the pathogenesis of cancer and cell cycle. In contrast, the low-risk group genes were significantly enriched in metabolism-related pathways such as complement and coagulation cascades, drug metabolism cytochrome p450, retinol metabolism, fatty acid metabolism, as well as linoleic acid metabolism (Fig. 9A). Meanwhile, the top 2 driver genes TP53 and CTNNB1 were significantly different between high (Fig. 9B) and low-risk groups (Fig. 9C).

### IC50 of chemotherapeutic drugs between the high- and low-risk group

**Fig. 10:**
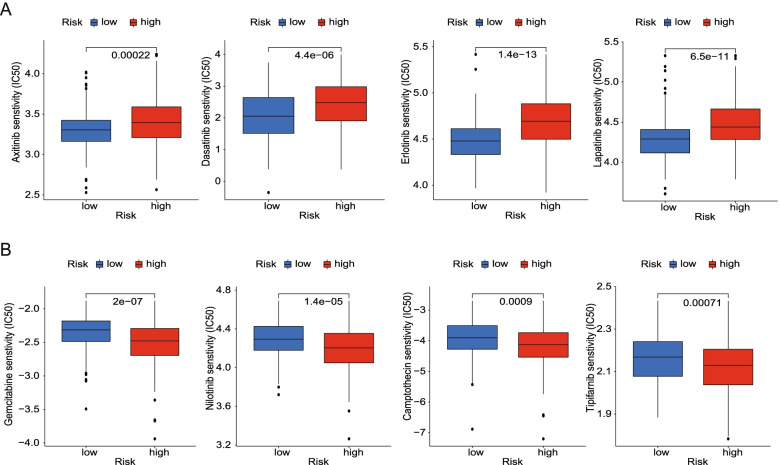
IC50 of chemotherapeutic drugs between the high- and low-risk group based on the pyroptosis-related genes signature in TCGA dataset. **A** Patients in the high-risk group were more sensitive to Axitinib, Dasatinib, Erlotinib, and Lapatinib (*p <* 0.001). **B** Patients in the low-risk group were more sensitive to Gemcitabine, Nilotinib, Camptothecin, and Tipifarnib (*p <* 0.001)

We further examined whether the risk score can predict the sensitivity of patients to chemotherapy, which showed that patients in the high-risk group were more sensitive to Axitinib, Dasatinib, Erlotinib, Lapatinib (*p* < 0.001) (Fig. 10A). The patients in the low-risk group were more sensitive to Gemcitabine, Nilotinib, Camptothecin, and Tipifarnib (p < 0.001) (Fig. 10B). These results suggested that PRGs were of great significance in targeted drug therapy.

### Validated PRGs between HCC tissues and adjacent Normal tissues

**Fig. 11:**
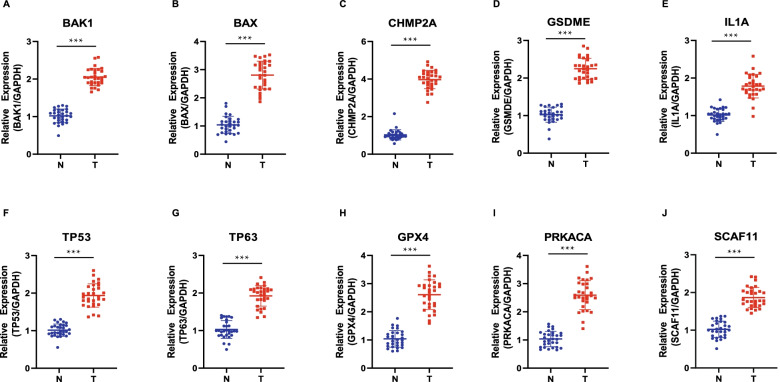
Validated PRGs between HCC tissues and adjacent tissues. **A**-**J** BAK1, BAX, CHMP2A, GSDME, IL1A, TP53, TP63, GPX4, PRKACA and SCAF11 in HCC tissues

To explore the expression of BAK1, BAX, CHMP2A, GSDME, IL1A, TP53, TP63, GPX4, PRKACA and SCAF11 in HCC tissues, we detected PRGs expression in HCC tissues from 30 patients by qRT-PCR assay. The results of qRT-PCR suggested that BAK1, BAX, CHMP2A, GSDME, IL1A, TP53, TP63, GPX4, PRKACA and SCAF11 were highly expressed in HCC tissues (Fig. 11A-J).

## Discussion

As a novel programmed cell death, pyroptosis played dual roles in the pathogenesis and treatment of several malignancies. It could promote the cancer cell apoptosis, which may serve as a treatment target for cancer [33]. In contrast, it could simulate the transformation of normal somatic cells to the cancer cells through releasing inflammatory factors [16]. Besides, it could regulate the cancer cell proliferation, invasion, migration and resistance to the chemotherapeutic agents, thereby affecting the tumor progression that was closely related to the patient prognosis [34]. Due to our understanding on the regulation of pyroptosis in the HCC is still limited. This led us to investigate the roles of pyroptosis in the HCC by establishing a predictive model for the prognosis of HCC.

In this study, we studied the mRNA expression of 52 currently known PRGs that had been well acknowledged to be expressed in HCC samples and normal tissues. Among these PRGs, 42 were differentially expressed. Then two clusters were generated by the consensus clustering analysis based on the DEGs. There were significant differences in clinical features including T stage, grade, gender, and stage among different clusters. KM curve analysis showed that cluster 1 had a better prognosis than cluster 2.

To date, our understanding on the correlation between PRGs and the survival time of HCC is still limited. In this study, we established a model for predicting OS of HCC patients based on 10 PRGs, including BAK1, BAX, CHMP2A, GSDME, IL1A, TP53, TP63, GPX4, PRKACA and SCAF11. In a previous study, Hu et al. suggested that single BAK or BAX, or BAK/BAX-caspase-3-GSDME pathway involved in the chemotherapy-induced pyroptosis, together with palmitoylation of GSDME [35]. Yu et al. showed that GSDME mediated lobaplatin-induced pyroptosis downstream of the ROS/JNK/Bax-mitochondrial apoptotic pathway and caspase-3/− 9 activation in colon cancer cellsx [9]. Zhang et al. demonstrated that miltirone inhibited HCC cells growth through BAX–caspase–GSDME-dependent pyroptotic by regulating ROS/mitogen-activated and extracellular signal-regulated kinase (MEK)/extracellular regulated protein kinases 1/2 (ERK1/2) pathway [36]. Hattori et al. provided evidence that CHMP2A depletion induced signaling complexes (iDISC)-mediated noncanonical Caspase-8 activation on immature autophagosomal membranes and inhibited tumor growth in a mouse xenograft model [37]. GSDME was identified as a pore forming molecule, which was activated following caspase-3-mediated cleavage resulting in so-called secondary necrosis following apoptotic cell death, or in primary necrotic cell death without an apoptotic phase [38]. Jiang et al. found that the caspase-3/GSDME signal pathway was a switch between apoptosis and pyroptosis in cancer [39]. Yu et al. suggested that cleavage of GSDME by caspase-3 determines lobaplatin-induced pyroptosis in colon cancer cells [9]. Zhang et al. demonstrated that miltirone induced cell death in hepatocellular carcinoma cell through GSDME dependent pyroptosis [36]. Lachner indicated that expression of pro-inflammatory IL1A, IL1B and pyroptotic pore-forming gasdermin (GSDM) D was downregulated during terminal differentiation of human keratinocytes in vitro. They screened pyroptosis-related protein families for members with predominant expression in the skin and provided evidence for normal keratinocyte differentiation-associated expression of specific IL1F cytokines and proteins related to pyroptosis [40]. Zhang et al. found that transcription factor p53 suppressed tumor growth by prompting pyroptosis in non-small-cell lung cancer [41]. N-terminal isoforms of p63 are TAp63 and ΔNp63 [42]. These findings suggested that lncRNA RP1-85F18.6 may trigger colorectal cancer cell proliferation, invasion and cell cycle disruption, and suppressed the apoptosis and pyroptosis of colorectal cancer cells through regulating ΔNp63 expression [43]. Zhu et al. suggested that GPx4, as a requisite gateway to both ferroptosis and pyroptosis, may serve as a critical molecular target for developing effective drugs for controlling infection and sepsis [44]. PRKACA was identified to be PRG and used to construct prognostic risk prediction models in colon adenocarcinoma and glioma [20, 45]. SCAF11 was identified to be PRG and used to construct prognostic risk prediction models in breast cancer [28].

To further assess the prognostic value of these PRGs, prognostic model constructed by 10 PRGs in the TCGA database was validated to perform well in external datasets (GSE14520 and ICGC-LIRI-JP). We revealed that the relationship between PRGs signature and immune infiltration by TIMER, CIBERSORT, CIBERSORT-ABS, QUANTISEQ, MCPCOUNTER, XCELL and EPIC algorithms [46]. GSEA revealed that the genes in the high-risk group of TCGA cohorts were significantly enriched in tumor and immune-related pathways. In contrast, the low-risk group genes were significantly enriched in metabolism-related pathways. Therefore, we speculate that our prognostic signature is related to tumor immunity and metabolism. Following that, we examined whether the risk score can predict the sensitivity of patients to chemotherapy and found that a low-risk score was linked to IC50 of chemotherapeutics such as Gemcitabine, Nilotinib, Camptothecin, Tipifarnib (*p* < 0.001), whereas a high-risk score was linked to Axitinib, Dasatinib, Erlotinib, Lapatinib (p < 0.001), implying that signature served as a prospective predictor for targeted drug therapy. Meanwhile, the top 2 driver genes TP53 and CTNNB1 were significantly different between high and low risk groups. Furthermore, PCR was given to detect the expression of PRGs expression in the tissue samples obtained from 30 HCC patients. The results of qRT-PCR suggested that BAK1, BAX, CHMP2A, GSDME, IL1A, TP53, TP63, GPX4, PRKACA and SCAF11 were highly expressed in HCC tissues.

Some related studies have been on pyroptosis-related gene signatures in HCC, which proved that PRGs played important roles in predicting the prognosis of HCC [6–8]. Unlike these studies, we firstly added mutation data analysis of PRGs, and the ICGC-LIRI-JP dataset to verify the validity of the model. Secondly, our data showed that PRGs signature were related to the sensitivity of chemotherapy. Finally, different algorithms such as TIMER, CIBERSORT, CIBERSORT-ABS, QUANTISEQ, MCPCOUNTER, XCELL and EPIC were used to analyze the infiltration of immune cells in high and low risk groups. Our study provides a novel gene signature for predicting the prognosis of HCC patients and offers a significant basis for future studies of the relationships between PRGs and immunity in HCC.

## Supplementary Information

**Additional file 1.**

**Additional file 2.**

**Additional file 3: Supplementary Fig. S1.** The expression and interaction of 42 PRGs. (A) PPI network showing the interactions of the PRGs (interaction score = 0.9). (B) The correlation network of the PRGs (red line: positive correlation; blue line: negative correlation. The depth of the colors reflects the strength of the relevance).

**Additional file 4: Supplementary Fig. S2.** The Volcano plots of DEGs between HCC tissues and adjacent tissues.

**Additional file 5: **S**upplementary Fig. S3.** The correlation of gene expression with each type of immune cell infiltration.

## Data Availability

All data included in this study are available upon request by contact with the corresponding author.

## Abbreviations

HCC: Hepatocellular carcinoma
TCGA: The cancer genome atlas
GEO: Gene expression omnibus
ICGC: International cancer genome consortium
FPKM: Fragments per kilobase million
DEG: Differential expression gene
FDR: False discovery rate
RS: Risk score
OS: Overall survival
ROC: Receiver operating characteristic
KM: Kaplan–meier
GSEA: Gene set enrichment analysis
HR: Hazard ration
95% CI: 95% confidence interval
AUC: Area under the curve
