# Supplementary figures and images for: Pyroptosis related genes signature predicts prognosis and immune infiltration of tumor microenvironment in hepatocellular carcinoma
Source: BMC Cancer. 2022 Sep 20;22:999. doi: 10.1186/s12885-022-10097-2 (PMC9491002; doi:10.1186/s12885-022-10097-2)

A

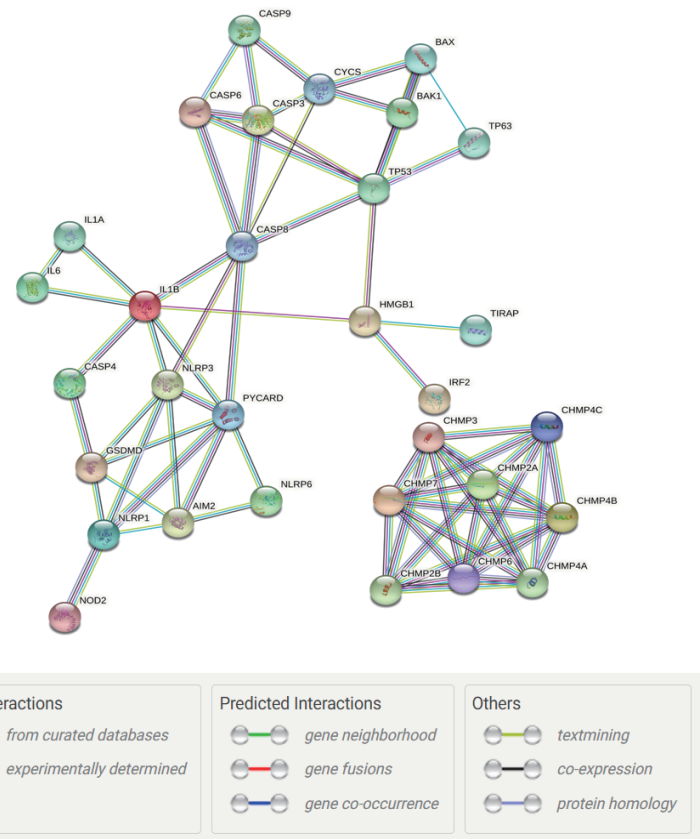

B

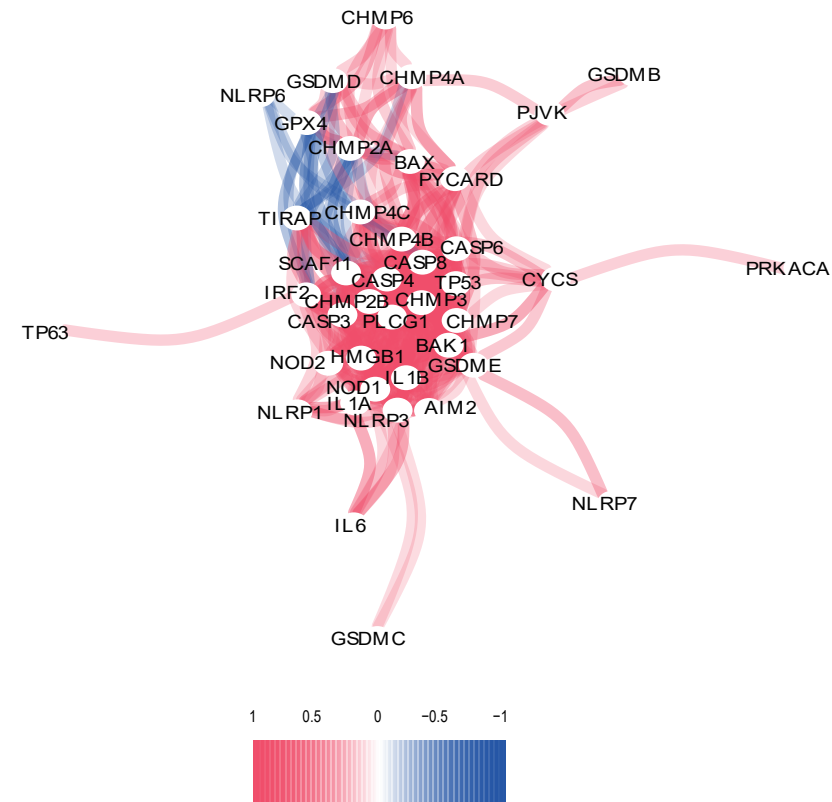

Supplement: Supplementary file 3 — Additional file 3: Supplementary Fig. S1. The expression and interaction of 42 PRGs. (A) PPI network showing the interactions of the PRGs (interaction score = 0.9). (B) The correlation network of the PRGs (red line: positive correlation; blue line: negative correlation. The depth of the colors reflects the strength of the relevance). [file 12885_2022_10097_MOESM3_ESM.pdf]

A

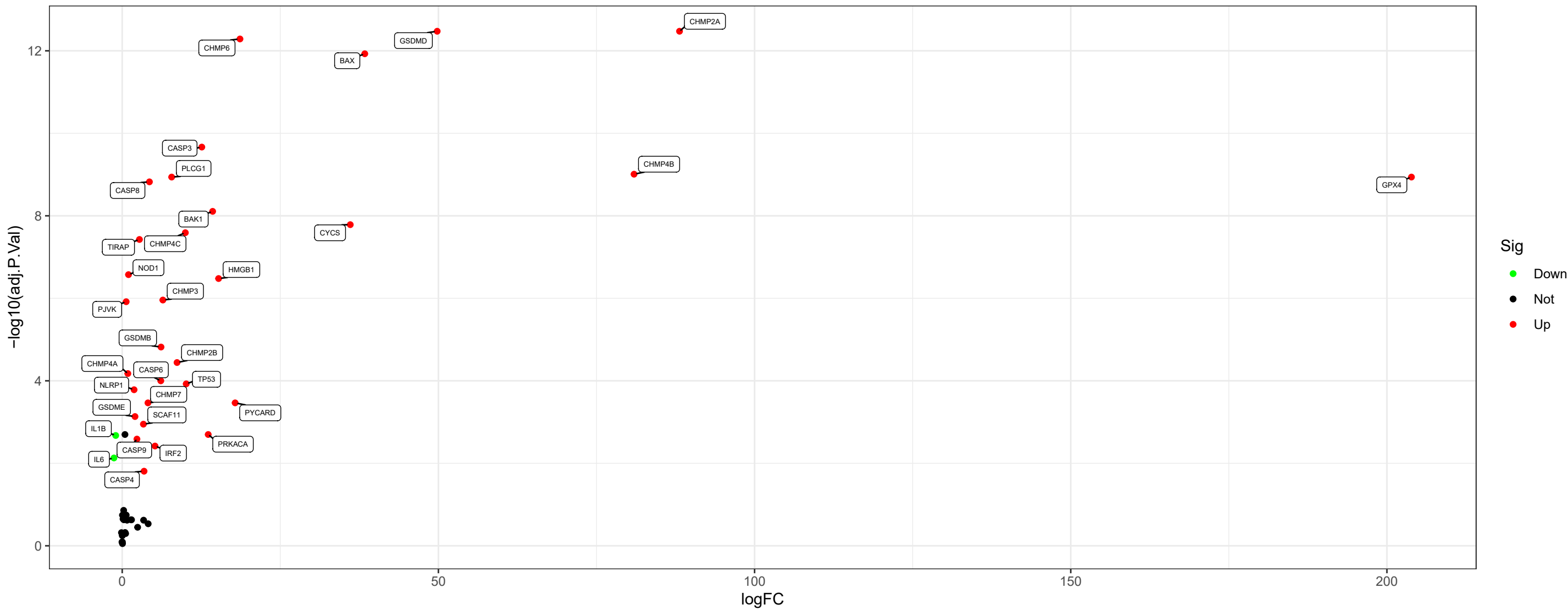

Supplement: Supplementary file 4 — Additional file 4: Supplementary Fig. S2. The Volcano plots of DEGs between HCC tissues and adjacent tissues. [file 12885_2022_10097_MOESM4_ESM.pdf]

A

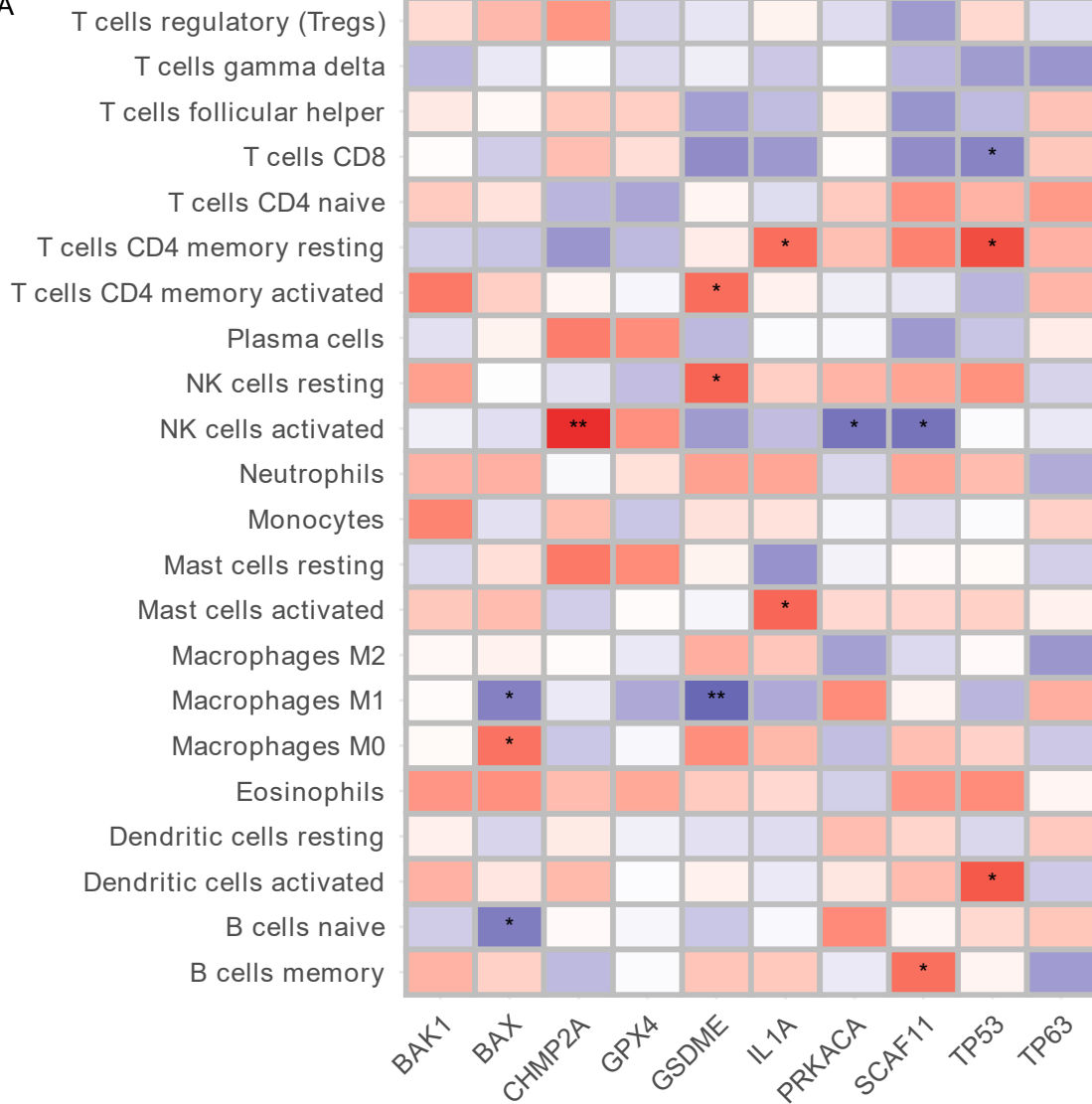

\*\*\* p&lt;0.001

\*\* p&lt;0.01

\* p&lt;0.05

Correlation

0.4

0.2

0.0

-0.2

Supplement: Supplementary file 5 — Additional file 5: Supplementary Fig. S3. The correlation of gene expression with each type of immune cell infiltration. [file 12885_2022_10097_MOESM5_ESM.pdf]
